# An Unusual Cause of Longstanding Diarrhea

**DOI:** 10.7759/cureus.52161

**Published:** 2024-01-12

**Authors:** Isairis Peralta, Elizabeth Hubbard, Laurentia Nodit

**Affiliations:** 1 Pathology, University of Tennessee Medical Center/University of Tennessee Graduate School of Medicine, Knoxville, USA

**Keywords:** colchicine toxicity, chronic diarrhea, weight loss, ring mitoses, colchicine poisoning

## Abstract

Colchicine is an alkaloid drug used in multiple medical conditions. It has a narrow therapeutic index, and gastrointestinal symptoms can occur at the beginning or after long-term therapy. Unintentional toxicity is common and has a high mortality rate when missed. Histopathologic recognition is challenging, and timely identification is conducted to improve patients' outcomes. We describe the case of a 77-year-old female who presented to the emergency room for dehydration, longstanding diarrhea, and weight loss. Upper and lower endoscopies showed erythematous mucosa without bleeding in the gastric antrum and an unremarkable duodenum and colon. Duodenal biopsies demonstrated partial villous atrophy with elongated glands and numerous arrested ring mitoses, consistent with colchicine toxicity.

## Introduction

Since ancient times, colchicine has been the staple treatment for gout disease. Its use in medical practice has survived centuries of close medical scrutiny, traveling the globe from Asia to America, with a continuously expanding list of disorders where it plays a leading therapeutic role [1]. The botanical compound is derived from the flower *Colchicum autumnale*, *Gloriosa superba* species [2]. In early literature, colchicine-based plants were mentioned mostly for their cathartic and poisonous effects and later for use in the treatment of pain and swelling [1]. In addition to its use in the prevention of gout disease, it is most commonly known for its anti-inflammatory activity in a large variety of disorders like familial Mediterranean fever, recurrent pericarditis [3], pseudo-gout, Behcet's disease, neutrophilic dermatoses [4], and its most recent investigation for the treatment of COVID-19 disease [5]. Gastrointestinal symptoms at the beginning of long-term therapy are well known; however, histopathologic findings have been recently reported [6], and early recognition can help with better patient outcomes.

Altogether, colchicine's low cost and effectiveness may provide an important addition to other standard therapies. The toxic effects of colchicine are serious even today, as there is no antidote and overdose carries a high mortality rate.

## Case presentation

A 77-year-old female with a clinical history significant for obesity, diabetes mellitus type 2, hyperlipidemia, peripheral artery disease, calcium pyrophosphate deposition ("pseudo-gout"), lumbar degenerative disc disease, and hypertensive renal disease presented to the emergency room with dehydration following a longstanding diarrheal illness. Her diarrhea started several months ago (after right shoulder replacement surgery) and was associated with abdominal pain, nausea, vomiting, decreased food intake, and unintentional 43-pound weight loss. The patient denied using antibiotics or any stool softeners. No blood was noticed in her stool. She had not been around other sick individuals, and her husband was not experiencing similar symptoms. The patient did not complain of any fever, chills, or shortness of breath and did not travel recently. She stated that she could typically tolerate breakfast in the morning but nothing else during the day. A *Clostridioides difficile* rapid test was negative, and CT of the abdomen and pelvis showed only fluid-filled large bowels with no other incidental findings. Her home medications included metformin, Tiadylt, Zofran, Norco, colchicine, clonidine, hydrochlorothiazide-lisinopril, venlafaxine, fish oil, and estradiol.

**Figure 1:**
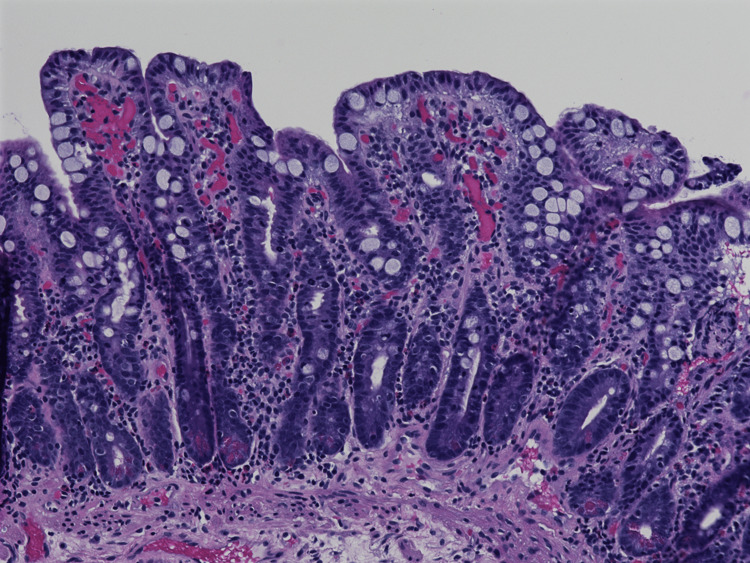
Microscopic photograph demonstrating villous blunting in the duodenum; 20x magnification H&E stain

**Figure 2:**
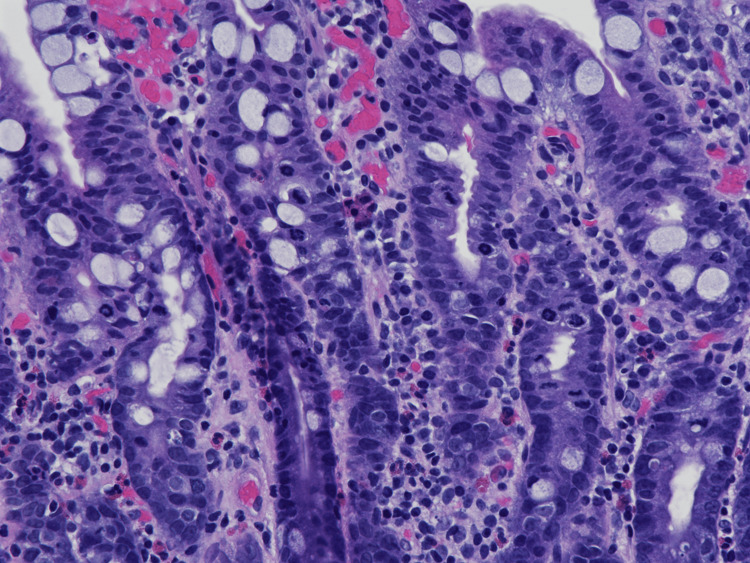
Microscopic photograph demonstrating increased arrested mitoses in the duodenal glands; 40x magnification H&E stain

Upper and lower endoscopies were performed, which revealed salmon-colored mucosa in the esophagus, erythematous mucosa without bleeding in the gastric antrum, and an unremarkable duodenum and colon. Biopsies were obtained. Histopathologic examination of the upper gastrointestinal tract epithelium was significant for the presence of numerous arrested mitoses; duodenal mucosal biopsies revealed partial villous atrophy (Figure 1), with elongated glands without increased intraepithelial lymphocytes or chronic inflammation but showed numerous arrested ring mitoses (Figure 2). The *Helicobacter pylori* immunostain was negative. By microscopic examination, the duodenal sample showed no parasites, acute inflammation, or malignancy. Serologic testing for celiac disease was negative. These findings were felt to be consistent with colchicine toxicity, given the patient’s clinical history.

## Discussion

The histologic identification of colchicine toxicity in gastrointestinal biopsies is difficult without the appropriate clinical context, as it has overlapping features with high-grade dysplasia, neoplasia, and Taxol toxicity [7] in the esophagus and celiac disease in the duodenum. In previous studies, mitotic figures arrested in metaphase (referred to as "ring" mitoses) and villous atrophy were described in the duodenal and antral biopsies of patients with clinical evidence of colchicine toxicity [6]. Other findings included epithelial pseudo-stratification, loss of polarity, and crypt apoptosis. The absence of intraepithelial lymphocytosis and increased chronic inflammation in the lamina propria, as well as negative serologic testing, suggest that celiac disease is less likely in this case.

Colchicine is predominantly metabolized in the gastrointestinal tract and has a narrow therapeutic index; at the beginning of therapy, more than 10% of the patients experience gastrointestinal side effects like diarrhea, nausea, abdominal pain, and vomiting. Unintentional toxicity is common and has a high mortality rate when missed. Colchicine exerts its action by reducing cellular cytoskeletal function, binding to tubulin, and disrupting its polymerization into microtubules. Through impairment of cellular protein assembly, it affects a variety of processes, resulting in decreased cellular motility, degranulation, migration, and mitosis arrest [8]. The anti-inflammatory function seems to be performed through suppression of myeloid-cell activation [5]. It is metabolized via cytochrome P450 3A4; therefore, coadministration with agents that inhibit this isoenzyme can produce elevated colchicine concentrations, resulting in severe and sometimes fatal adverse events [9].

At the time of the biopsy interpretation, our patient was taking colchicine (0.6 mg daily), which was prescribed long-term to reduce the risk of recurrent attacks of her calcium pyrophosphate deposition disease. The gastroenterologist was notified about the pathology findings, and colchicine was stopped. The patient’s symptoms had ceased, and she was gaining weight upon subsequent follow-ups (at one and five months) after drug discontinuation. Currently, the patient is doing well.

## Conclusions

A large variety of drugs (prescription and non-prescription), herbs, and dietary supplements can cause diarrhea as a side effect. A review of the patient's medication list is important. Appropriate recognition of clinical symptoms and histopathologic findings of colchicine toxicity is crucial for adequate patient management.
